# Deficiency of Senescence Marker Protein 30 Exacerbates Cardiac Injury after Ischemia/Reperfusion

**DOI:** 10.3390/ijms17040542

**Published:** 2016-04-11

**Authors:** Shinpei Kadowaki, Tetsuro Shishido, Toshiki Sasaki, Takayuki Sugai, Taro Narumi, Yuki Honda, Yoichiro Otaki, Daisuke Kinoshita, Tetsuya Takahashi, Satoshi Nishiyama, Hiroki Takahashi, Takanori Arimoto, Takuya Miyamoto, Tetsu Watanabe, Akihiko Ishigami, Yasuchika Takeishi, Isao Kubota

**Affiliations:** 1 Department of Cardiology, Pulmonology, and Nephrology, Yamagata University School of Medicine, 2-2-2 iidanihi, Yamagata 990-9585, Japan; s-kadowaki@med.id.yamagata-u.ac.jp (S.K.); toshikisasaki@yahoo.co.jp (To.S.); t-sugai@med.id.yamagata-u.ac.jp (Ta.S.); t-narumi@med.id.yamagata-u.ac.jp (T.N.); y-honda@med.id.yamagata-u.ac.jp (Y.H.); y-otaki@med.id.yamagata-u.ac.jp (Y.O.); d-kinoshita@med.id.yamagata-u.ac.jp (D.K.); t.tetsuya@med.id.yamagata-u.ac.jp (T.T.); mnisiyam@med.id.yamagata-u.ac.jp (S.N.); hitakaha@med.id.yamagata-u.ac.jp (H.T.); t-arimoto@med.id.yamagata-u.ac.jp (T.A.); tamiyamo@med.id.yamagata-u.ac.jp (T.M.); tewatana@med.id.yamagata-u.ac.jp (T.W.); ikubota@med.id.yamagata-u.ac.jp (I.K.); 2 Molecular Regulation of Aging, Tokyo Metropolitan Institute of Gerontology, 35-2 Sakae-cho, Itabashi-ku, Tokyo 173-0015, Japan; ishigami@timig.or.jp; 3 Department of Cardiology and Hematology, Fukushima Medical University, 1 Hikarigaoka, Fukushima 960-1295, Japan; takeishi@fmu.ac.jp

**Keywords:** ischemia reperfusion, SMP30, cardiomyocyte, GSK-3β

## Abstract

Early myocardial reperfusion is an effective therapy but ischemia/reperfusion (I/R) causes lethal myocardial injury. The aging heart was reported to show greater cardiac damage after I/R injury than that observed in young hearts. Senescence marker protein 30 (SMP30), whose expression decreases with age, plays a role in reducing oxidative stress and apoptosis. However, the impact of SMP30 on myocardial I/R injury remains to be determined. In this study, the left anterior descending coronary artery was occluded for 30 min, followed by reperfusion in wild-type (WT) and SMP30 knockout (KO) mice. After I/R, cardiomyocyte apoptosis and the ratio of infarct area/area at risk were higher, left ventricular fractional shortening was lower, and reactive oxygen species (ROS) generation was enhanced in SMP30 KO mice. Moreover, the previously increased phosphorylation of GSK-3β and Akt was lower in SMP30 KO mice than in WT mice. In cardiomyocytes, silencing of SMP30 expression attenuated Akt and GSK-3β phosphorylation, and increased Bax to Bcl-2 ratio and cardiomyocyte apoptosis induced by hydrogen peroxide. These results suggested that SMP30 deficiency augments myocardial I/R injury through ROS generation and attenuation of Akt activation.

## 1. Introduction

Percutaneous coronary intervention has been reported to reduce the mortality in patients with acute coronary infarction, since coronary reperfusion therapy is the most effective treatment for salvaging viable myocardium [1,2]. Ischemia/reperfusion (I/R) involves reactive oxygen species (ROS) generation, intracellular Ca^2+^ disturbance, rapid pH restoration, and inflammation [3]; therefore, I/R is accompanied by detrimental manifestations [3], including myocardial necrosis and apoptosis [4,5]. Apoptotic cell death contributes to an increase in infarct size; inhibition of this component contributes to an improved cardiac function [6]. Several experimental animal models have provided new targets to protect the heart against I/R injury; however, several clinical trials failed to report any protective effects against myocardial I/R injury [7,8,9]. Moreover, it is expected that the elderly population continues to increase rapidly and will reach 35.8 million (27%) in 2055 [10]. Elderly patients have worse clinical outcomes than the non-elderly patients after percutaneous coronary interventions [11]. In animal models, aging hearts showed worse I/R injury than in young hearts [12,13]. Previous studies have reported that abnormalities in mitochondrial function and calcium handling, excessive generation of oxidative stress, and attenuation of cardioprotective signaling are potentially implicated in the aging heart [14,15,16]. However, the precise mechanism by which cardiac senescence induces a loss of protective function against I/R injury is not fully understood.

It has been reported that the expression of senescence marker protein 30 (SMP30) decreases with aging in an androgen-independent manner; this mechanism is also observed in the heart [17,18]. SMP30 plays multifunctional roles in cell regulation and is expressed in most organs [18,19]. Overexpression of SMP30 decreases tumor necrosis factor-α- or lipopolysaccharide-induced apoptosis in the liver, and suppresses oxidative stress in the brain and lungs [20,21]. Moreover, SMP30 KO mice show an accelerated senescence in the kidneys, worsening of glucose intolerance [22], and abnormal mitochondria in liver cells [23]. There is no significant difference in the cardiac function between WT mice and SMP30 KO mice, however; we have recently reported that SMP30 plays a protective role in angiotensin II-induced cardiac hypertrophy and doxorubicin-induced cardiotoxicity in mice via anti-apoptosis and anti-oxidant effects [17,24,25]. However, the protective role of SMP30 in myocardial I/R injury has not been clearly demonstrated. In the present study, we evaluated the mechanisms by which SMP30 deficiency exacerbates myocardial I/R injury.

## 2. Results

### 2.1. Deficiency of SMP30 Exacerbates Infarct Size Induced by I/R

**Figure 1:**
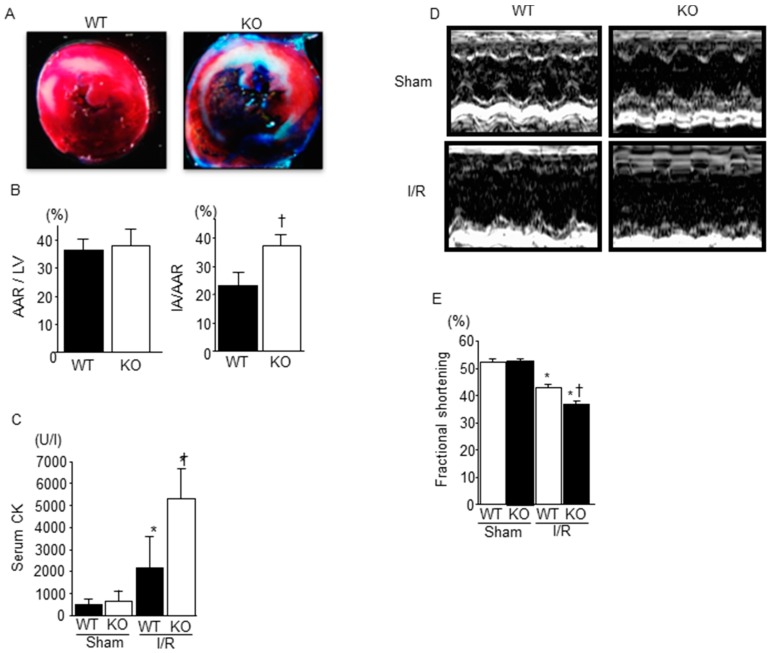
Comparison of myocardial I/R injury between WT and SMP30 KO miceL (**A**) Representative photomicrographs of Evans blue/TTC-stained hearts obtained from WT and SMP30 KO mice subjected to 30 min of ischemia and 24 h of reperfusion. The TTC red-stained area indicates AAR and the unstained area indicates IA; (**B**) Quantitative analysis of AAR/LV and IA/AAR. Graphs show mean ± SE of AAR/LV and IA/AAR (*n* = 7). ^†^ *p* < 0.05 *vs.* WT I/R mice; (**C**) Serum CPK levels 24 h after I/R or sham operation in WT mice and SMP30 KO mice. Graphs show mean ± SE of serum CPK levels (*n* = 7). I/R, ischemia/reperfusion; AAR, area at risk; IA, infarct area; CPK, creatine phosphokinase. * *p* < 0.05 *vs.* sham-operated mice, ^†^ *p* < 0.05 *vs.* WT I/R mice; (**D**) Representative M-mode echocardiograms of left ventricles in WT and SMP30 KO mice 24 h after I/R and sham operation; (**E**) Fractional shortening 24 h after I/R or sham operation in WT mice and SMP30 KO mice. Graphs show mean ± SE of fractional shortening (*n* = 10 to 15). * *p* < 0.05 *vs.* sham-operated mice, ^†^ *p* < 0.05 *vs.* WT I/R mice.

We compared myocardial damage in WT mice and SMP30 KO mice. After 30 min of ischemia and 24 h of reperfusion, the area at risk (AAR)/LV ratio in SMP30 KO mice was similar to that in WT mice. However, the size of infarction area (IA)/AAR ratio was significantly higher in SMP30 KO than in WT mice (Figure 1A,B). In addition, serum CPK levels of SMP30 KO mice were significantly higher than those of WT mice 24 h after I/R (Figure 1C). There was no significant difference in serum CPK levels between sham-operated WT and SMP30 KO mice. These results indicate that SMP30 deficiency enhanced myocardial injury during I/R.

### 2.2. Cardiac Function after I/R Was Exacerbated in SMP30 KO Mice Compared with WT Mice

We performed an echocardiography to assess the cardiac function in SMP30 KO and WT mice. There were no significant changes in echocardiographic parameters between sham-operated SMP30 KO and WT mice (Table 1, Figure 1D). At 24 h after reperfusion, the fractional shortening was significantly lower in SMP30 KO mice than in WT mice as shown in Table 1 and Figure 1E.

### 2.3. Effect of SMP30 Deficiency on Oxidative Stress and Myocardial Apoptosis Induced by I/R

**Figure 2:**
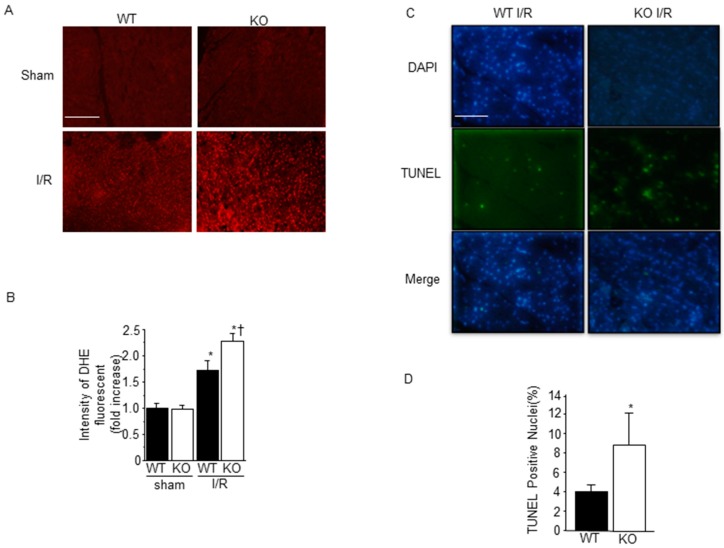
Increased myocardial apoptosis and ROS generation in SMP30 KO mice: (**A**) Representative images of DHE staining of frozen left ventricular tissues 30 min after I/R or sham operation in SMP30 KO and WT mice; (**B**) Quantitative analysis of intensity of DHE fluorescent. Data are reported as mean ± SE (*n* = 6). DHE, dihydroethidium. Scale bars: 100 μm. * *p* < 0.05 *vs.* sham-operated mice, and ^†^ *p* < 0.05 *vs.* WT I/R mice; (**C**) Representative TUNEL-stained sections from WT and SMP30 KO mice subjected to 30 min of ischemia and 24 h of reperfusion or sham operation; (**D**) Percentages of TUNEL-positive nuclei in sections from WT and SMP30 KO mice. Data are reported as mean ± SE (*n* = 6). TUNEL, TdT-mediated dUTP nick end-labeling. Scale bars: 200 μm. * *p* < 0.05 *vs.* sham-operated mice.

Because we have previously shown that ROS generation was increased in SMP30 KO mice after angiotensin II or doxorubicin administration, we evaluated the myocardial oxidative stress after I/R by dihydroethidium (DHE), which indicates superoxide production. I/R increased ROS generation in both WT and SMP30 KO mice; however, ROS generation induced in I/R SMP30 KO mice was significantly higher than that in WT mice (Figure 2A,B). Since ROS generation during I/R induced myocardial apoptosis, we performed a TUNEL staining to investigate the extent of apoptosis in the ischemic area after I/R. TUNEL-positive cells were evident in heart sections obtained from both SMP30-KO and WT ischemic areas. There was no significant difference between WT mice and SMP30 KO mice in sham operation. SMP30 KO mice exhibited significantly higher percentages of TUNEL-positive nuclei compared with WT mice, as shown in Figure 2C,D. These results indicated that SMP30 deficiency enhanced ROS generation in the heart and cardiomyocyte apoptosis during I/R.

### 2.4. Phosphorylation of ERK1/2, Akt, and Glycogen Synthase Kinase-3β (GSK-3β) after I/R

**Figure 3:**
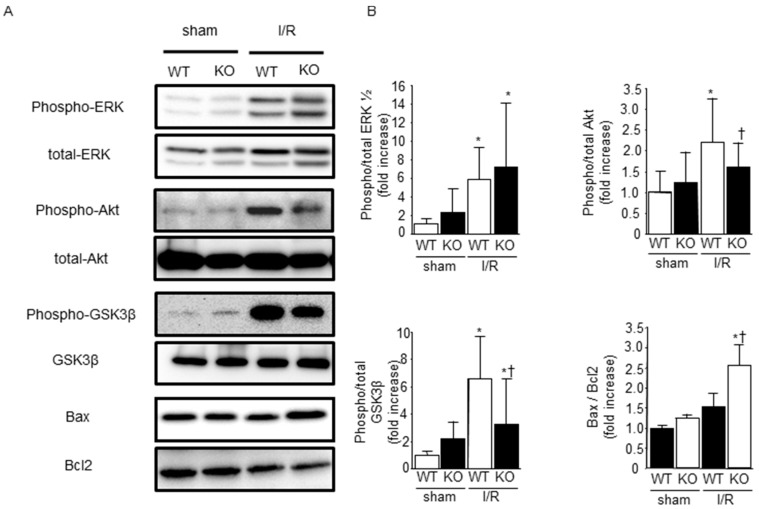
Effect of SMP30 deficiency on survival signaling proteins: (**A**) representative Western blots from WT and SMP30 KO mice subjected to 30 min of ischemia and 30 min of reperfusion; and (**B**) quantitative and statistical analyses of Western blots are shown. Data are represented as mean ± SE (*n* = 8). ERK, extracellular regulated kinase, 1/2; GSK-3β, glycogen synthase kinase-3β. * *p* < 0.05 *vs.* sham-operated mice, and ^†^ *p* < 0.05 *vs.* WT I/R mice.

Activation of the reperfusion injury salvage kinase (RISK) pathway contributes to the reduction of I/R injury [26,27]. Therefore, we examined the phosphorylation of ERK1/2, Akt, and GSK-3β before and after I/R. Western blots revealed that ERK1/2, Akt, and GSK-3β phosphorylation were increased 30 min after reperfusion. There was no significant difference in ERK1/2 phosphorylation between WT and SMP30 KO mice; however, phosphorylation of Akt and GSK-3β after I/R was suppressed in SMP30 KO mice compared with WT mice. Since Akt and GSK-3β regulate the myocardial apoptotic pathway, we evaluated Bax and Bcl-2 expression levels after ischemia/reperfusion. The ratio of Bax to Bcl-2 expression was significantly higher in SMP30 KO mice than in WT mice (Figure 3A,B).

### 2.5. SMP30 Silencing on ERK1/2, Akt, and GSK-3β Phosphorylation in Cardiomyocytes after H_2_O_2_ Stimulation

**Figure 4:**
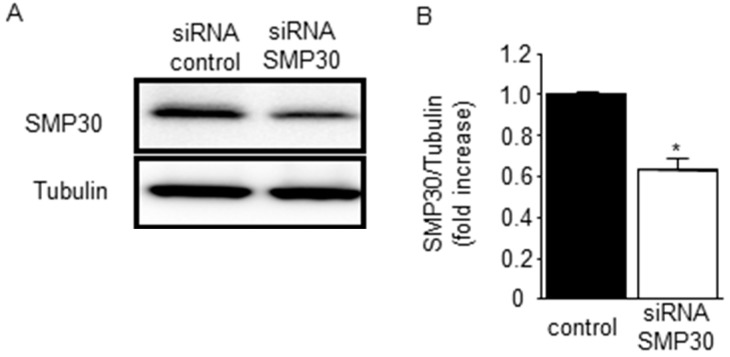
Transfection with siRNA targeting SMP30 in cardiomyocytes: (**A**) nonspecific control siRNA or siRNA targeting SMP30 were transfected into neonatal rat cardiomyocytes, and SMP30 expression was analyzed by Western blotting; (**B**) Expression of SMP30 was knocked down by its siRNA. Data are represented as mean ± SE (*n* = 4), * *p* < 0.05 *vs.* control siRNA group.

We found that Akt and GSK-3β phosphorylation were attenuated in SMP30-KO mice; however, it was possible that the potentiated generation of ROS in SMP30-KO mice after I/R (Figure 2A,B) influenced the degree of phosphorylation. To investigate the effect of SMP30, we transfected SMP30 siRNA or nonspecific control siRNA into neonatal rat cardiomyocytes. Expression of SMP30 was knocked down by its siRNA (Figure 4).

**Figure 5:**
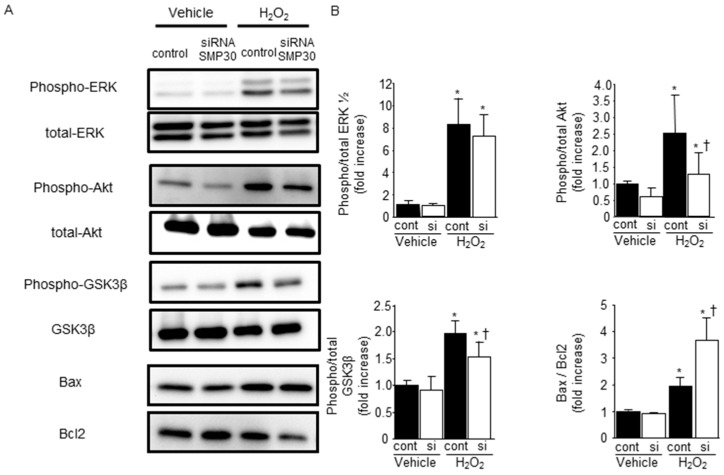
Effect of SMP30 silencing on survival signaling and apoptosis-associated proteins in cardiomyocytes: (**A**) representative Western blots from SMP30 siRNA and control siRNA cardiomyocytes subjected to 1 h of H_2_O_2_ stimulation; (**B**) Silencing SMP30 attenuated phosphorylation of Akt and GSK-3β and increased the ratio of Bax to Bcl-2 expression. Quantitative and statistical analyses of Western blots are shown. Data are represented as mean ± SE (*n* = 8), * *p* < 0.05 *vs.* vehicle control siRNA, and ^†^ *p* < 0.05 *vs.* H_2_O_2_ control.

To confirm the effect of SMP30 silencing on Akt and GSK-3β phosphorylation, neonatal cardiomyocytes were subjected to hydrogen peroxide (H_2_O_2__)_ stimulation. Phosphorylation of ERK1/2, Akt, and GSK-3β were increased after 1 h of H_2_O_2_ stimulation. SMP30 silencing significantly suppressed Akt and GSK-3β phosphorylation, but did not influence ERK1/2 phosphorylation (Figure 5A,B). Moreover, the ratio of Bax to Bcl-2 expression was significantly higher in SMP30 siRNA-transfected cardiomyocytes 24 h after H_2_O_2_ stimulation (Figure 5A,B).

### 2.6. Impact of Silencing of SMP30 Expression on Cardiomyocyte Apoptosis

**Figure 6:**
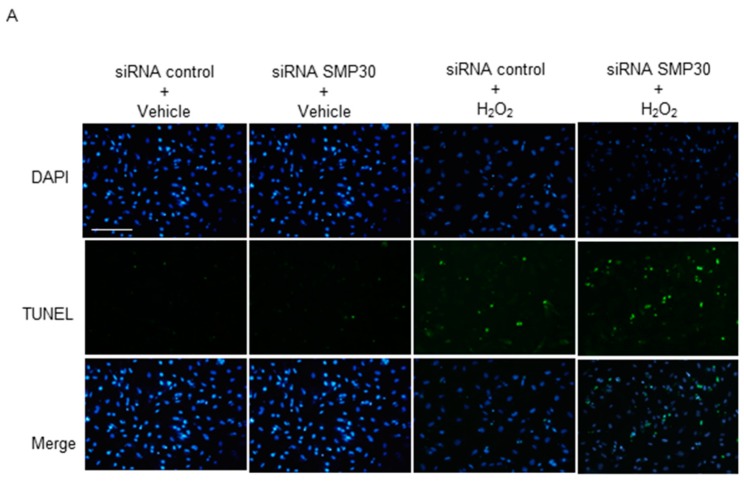

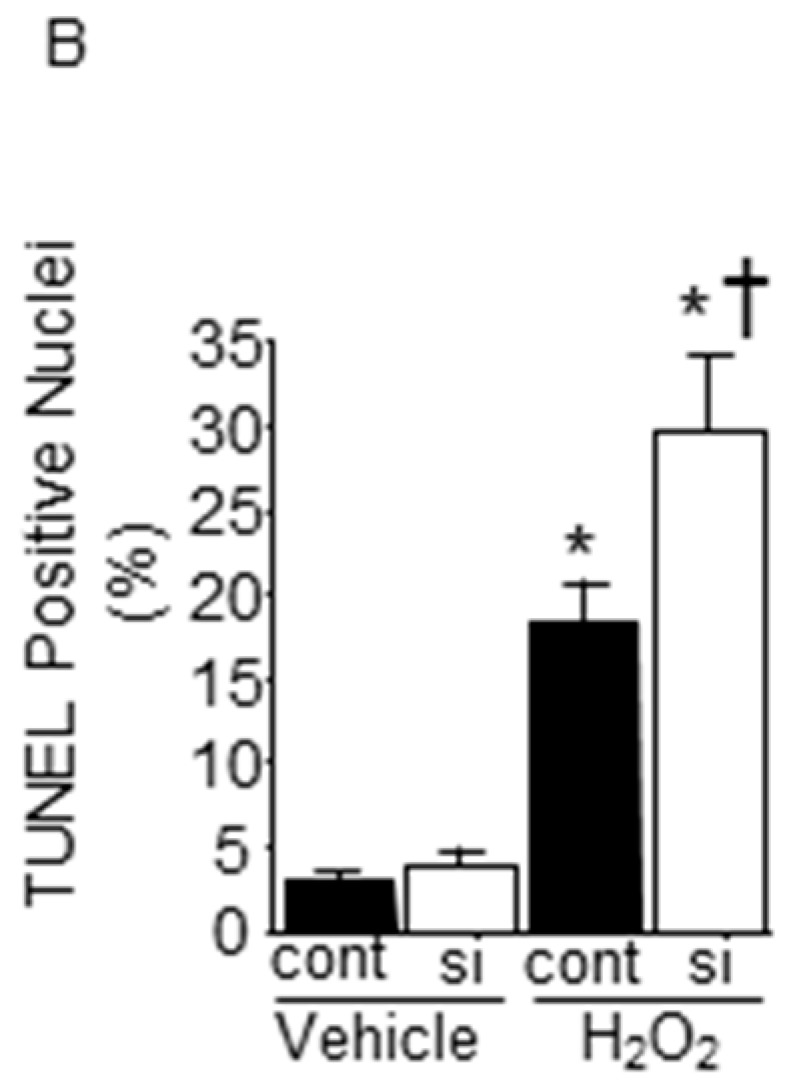
Evaluation of apoptosis induced by H_2_O_2_ stimulation *in vitro*: (**A**) representative TUNEL stained sections from SMP30 siRNA and control siRNA cardiomyocytes subjected to 24 h of H_2_O_2_ stimulation; (**B**) percentages of TUNEL-positive nuclei in sections. Data are represented as mean ± SE (*n* = 6), Scale bars: 200 μm, * *p* < 0.05 *vs.* vehicle control siRNA, and ^†^ *p* < 0.05 *vs.* H_2_O_2_ control siRNA.

SMP30 silencing inhibited ROS-mediated phosphorylation of Akt and GSK-3β in cardiomyocytes. Therefore, we examined TUNEL staining to confirm the effect of SMP30 deficiency on ROS-mediated cardiomyocyte apoptosis. We observed that H_2_O_2_ stimulation increased TUNEL-positive cardiomyocytes. Furthermore, the number of TUNEL-positive nuclei after H_2_O_2_ stimulation was significantly higher in SMP30 siRNA than in control siRNA cardiomyocytes (Figure 6A,B).

## 3. Discussion

The main findings of this study are as follows: (1) SMP30 KO exacerbated infarct size and LV dysfunction after I/R; (2) ROS generation and apoptosis after I/R in SMP30 KO mice were significantly higher than those in WT mice; (3) Downregulation of SMP30 attenuated the phosphorylation of Akt and GSK-3β after I/R or H_2_O_2_ stimulation; and (4) SMP30-downregulated cardiomyocytes showed increased susceptibility to H_2_O_2-_induced apoptosis.

SMP30 is reported to act as an anti-aging factor and prevents oxidative stress and apoptosis [25,28,29]. Advanced age worsens I/R injury due to abnormalities in mitochondrial function, oxidative stress, and increased susceptibility to apoptosis and necrosis [15,16]. In our study, infarct size after I/R was larger and LVFS was lower in SMP30 KO mice than in WT mice. In addition, SMP30 KO mice were far more susceptible to I/R-induced apoptosis associated with an increase in the Bax/Bcl-2 ratio. Age-associated SMP30 decrease might be related to the susceptibility of aging hearts to I/R injury.

ROS-induced ROS-release phenomenon occurs under excessive oxidative stress such as I/R injury [30]. ROS generation and apoptosis after I/R in SMP30 KO mice were significantly higher than that in WT mice. A previous study reported that catalase and superoxide dismutase activities were not significantly different between WT and SMP30 KO mice, suggesting that SMP30 antioxidant effect may result from suppressing ROS generation rather than scavenging ROS [31]. Previous study reported that ascorbic acid deficiency affected Nrf2 and NQO1 [32]. In this study, there is no significant difference in ascorbic acid between WT and SMP30KO mice. Therefore, Nrf2 and NQO1 might not be related in this study. However, we need to elucidate the role of SMP30 in the expression and activity of Nrf2, NQO1, and SREBP1 in ischemia/reperfusion injury in the heart. SMP30 was reported to be associated with intracellular calcium homeostasis, which regulates ROS production in the mitochondria, through the sarcoplasmic reticulum Ca^2+^-ATPase (SERCA) in the kidneys, liver, and brain [33,34]. Therefore, it is also possible that SMP30 knockdown reduces SERCA levels and activity in the heart after I/R. This hypothesis was potentiated by the fact that SMP30 regulated SERCA activity, and that SMP30 KO mice demonstrated increases ROS generation due to calcium overload in the mitochondria [35,36].

The RISK pathway is a signaling cascade involving prosurvival kinases, which contribute to cardioprotection when activated during the time of reperfusion [27,37]. The phosphatidylinositol-3 kinase (PI3K), Akt, and ERK1/2 are involved in the RISK pathway. We found that phosphorylation of Akt and GSK-3β was lower in SMP30 KO mice than in WT mice. Similarly, H_2_O_2_-induced phosphorylation of Akt and GSK-3β was also inhibited by silencing of SMP30 expression in cardiomyocytes, suggesting that ROS-mediated activation of Akt and GSK-3β was at least in part SMP30-dependent. In the present study, H_2_O_2_ stimulation increased the Bax/Bcl-2 expression ratio and enhanced cardiomyocyte apoptosis by siRNA-mediated SMP30 silencing. Taken together, decreased phosphorylation of GSK-3β in SMP30-silenced cardiomyocytes also contributed to exacerbate cardiomyocyte apoptosis.

Based on the previous studies and our data, we suspect that SMP30 decreased expression in the heart of elderly patients is the cause of higher risk and worsened clinical outcomes during myocardial reperfusion. Lower expression of SMP30 in elderly patients induces increased ROS generation and attenuates AKT and GSK3β, both of which are associated with cardiomyocyte apoptosis. Therefore, preservation of SMP30 expression in aging heart is one of the possible therapeutic targets to inhibit cardiac dysfunction after myocardial infarction in elderly patients. To confirm this hypothesis, we need to evaluate cardiac function, cardiomyocyte apoptosis, and infarct size after ischemia/reperfusion in cardiac-specific overexpression of SMP30 mice in the future study.

There are several limitations in this study. We showed that deficiency of SMP30 exacerbates myocardial I/R injury; however, we did not show that endogenous or exogenous SMP30 has a protective role in myocardial I/R injury. To confirm this point, we might need to evaluate in a future study the cardioprotective role of SMP30 overexpression after I/R and H_2_O_2_ stimulation.

## 4. Experimental Section

### 4.1. Animal Protocol

SMP30 KO (C57BL/6 background) mice were established as previously reported [28]. Vitamin C water (1.5 g/L) was provided for the SMP30 KO mice, as described previously [17,24], because SMP30 is a gluconolactonase, a pivotal enzyme for ascorbic acid (vitamin C) synthesis [38]. Mice were housed in a pathogen-free facility with a 12:12 h light–dark cycle and were given free access to water and standard rodent chow. All experimental procedures were performed according to the animal welfare regulations of Yamagata University School of Medicine, and the study protocol was approved by the Animal Subjects Committee of Yamagata University School of Medicine (No. 21045, 16 March 2009). The investigation conformed to the Guide for the Care and Use of Laboratory Animals published by the National Institutes of Health.

### 4.2. Echocardiography

The cardiac function was evaluated by two-dimensional echocardiography with the use of an FF Sonic 8900 (Fukudadenshi Co., Tokyo, Japan) equipped with a 13-MHz phase-array transducer, at 24 h after I/R or sham operation. Mice were anesthetized with intraperitoneal administration of pentobarbital (35 mg/kg) so as not to compromise respiration and hemodynamic conditions. The internal dimensions of the left ventricle at end-diastole (LVEDD) and at end-systole (LVESD) were measured and averaged from three cardiac cycles. LV fractional shortening (% FS) was calculated as [(LVEDD − LVESD)/LVEDD] × 100 [26].

### 4.3. Heart Surgery of I/R Model

SMP30 KO and WT mice (age 10–12 weeks old) were anesthetized with an intraperitoneal administration of pentobarbital (100 mg/kg), orally intubated, and ventilated with a rodent respirator (Harvard Apparatus, Holliston, MA, USA). Inducing ischemia reperfusion and evaluating infarct size were performed as we previously described [26], using 5% Evans blue (Sigma Chemical Co., St. Louis, MO, USA) and 1% 2,3,5-triphenyltetrazolium chloride (TTC, Sigma Chemical Co.). Rrisk area and infarct area were measured using a Scion imaging system (Scion, Frederick, MD, USA) as we previously described [26]. Serum creatine phosphokinase (CPK) levels were measured in blood samples from mice 24 h after reperfusion. Serum CPK assay was performed with DRIKEM and CPK kits (FUJIFILM, Tokyo, Japan).

### 4.4. Western Blot Analysis

Samples were lysed in ice-cold lysis buffer and proteins were extracted as previously reported [39,40]. Protein concentrations were determined by protein assay (Bio-Rad Laboratories, Inc., Hercules, CA, USA). Equal amounts of proteins were subjected to 10% or 14% sodium dodecyl sulfate-polyacrylamide gel electrophoresis and transferred to polyvinylidene difluoride membranes. Immunoreactive bands were detected by using an ECL kit (Amersham Biosciences, Piscataway, NJ, USA). Membranes were incubated with the following primary antibodies: anti-phospho ERK1/2, anti-total ERK1/2, anti-phospho S473-Akt, total-Akt, anti-phospho GSK-3β, GSK-3β, anti-Bax, anti-Bcl-2, β-tubulin (Cell Signaling Technology, Inc., Danvers, MA, USA), and anti-SMP30 antibodies (SHIMA Laboratories Co., Ltd., Tokyo, Japan). To quantify the protein levels, the same membranes were reprobed with total proteins. The relative amount of phosphorylated proteins *vs.* total proteins was used for phosphorylation kinase activity. Ubiquitously expressed β-tubulin was used as a loading control.

### 4.5. Detection of Apoptosis

Twenty-four hours after I/R, the hearts were excised, fixed with a 10% solution of formalin in phosphate-buffered saline, embedded in paraffin, and serially cut from the apex to the base. Samples were stained by 4′,6-diamidino-2-phenulindole and TdT-mediated dUTP nick end-labeling (TUNEL) according to the manufacturer instructions. The percentage of TUNEL-positive cells was determined by counting 10 random fields per section under a microscope (BX50, Olympus, Tokyo, Japan). TUNEL staining was performed with a commercially available kit for the detection of end-labeled DNA according to the manufacturer instructions (Roche Applied Science, Tokyo, Japan).

In the *in vitro* study, cultured cardiomyocytes were fixed with 4% paraformaldehyde 24 h after H_2_O_2_ stimulation. These samples were stained with a TUNEL kit, and a 4′,6-diamidino-2-phenulindole (DAPI) staining was performed to normalize the results to the cell number. Approximately 400–600 nuclei from random fields were analyzed for each sample [41].

### 4.6. Assessment of Superoxide Generation

Thirty minutes after reperfusion, the heart tissues were embedded in optimum cutting temperature compound and sectioned at 3-μm thickness. Sections were incubated with 10 μM dihydroethidium (Sigma-Aldrich Co., St. Louis, MO, USA) at 37 °C for 30 min [17,42]. The mean dihydroethidium fluorescence intensity of the myocardium was quantified in 10 randomly selected fields in each section with the NIH Image J software under a microscope (BX50, Olympus, Tokyo, Japan).

### 4.7. Cultured Neonatal Rat Cardiomyocytes

Promptly after euthanasia by decapitation, hearts were collected from 1- to 2-day-old neonatal rat pups, and primary cultures of neonatal rat cardiomyocytes were performed as described previously [43,44]. Cardiomyocytes were kept in fetal bovine serum-supplemented Dulbecco’s modified Eagle medium (DMEM). SMP30 small interfering RNA (siRNA) was purchased from Thermo Scientific Dharmacon (Lafayette, CO, USA). Two days after cell seeding in culture medium, SMP30 siRNA was transfected into cardiomyocytes using Lipofectamine 3000 (Invitrogen, Carlsbad, CA, USA) according to the manufacturer instructions [39]. After serum starvation for 4 h, cardiomyocytes were stimulated with H_2_O_2_ (200 μM) for 1 and 24 h.

### 4.8. Statistical Analysis

All values are reported as mean ± standard error (SE) in figures and mean ± standard deviation (SD) in the table. Differences between groups were evaluated by analysis of variance (ANOVA) with Bonferroni test. A probability value less than 0.05 was considered statistically significant. The statistical analysis was performed with a standard statistical program package (JMP version 8; SAS Institute, Inc., Cary, NC, USA).

## 5. Conclusions

In this study, we found that SMP30 deficiency exacerbated myocardial I/R injury through an increase in oxidative stress and cardiomyocyte apoptosis. Moreover, a lower SMP30 expression suppressed Akt and GSK-3β phosphorylation and increased cardiomyocyte apoptosis after H_2_O_2_ stimulation. Therefore, a decreased SMP30 expression in the heart of elderly patients might be one of the causes of a poor prognosis after myocardial infarction.

## Figures and Tables

**Table 1:** Comparison of echocardiographic data after sham or I/R operation.

Echocardiografic Parameter	WT Sham	SMP30 KO Sham	WT I/R	SMP30 KO I/R
IVSd (mm)	0.75 ± 0.05	0.73 ± 0.06	0.73 ± 0.05	0.73 ± 0.04
LVPWd (mm)	0.71 ± 0.03	0.72 ± 0.07	0.74 ± 0.05	0.71 ± 0.04
LVEDd (mm)	3.01 ± 0.11	3.08 ± 0.08	2.92 ± 0.31	2.87 ± 0.32
LVESd (mm)	1.42 ± 0.07	1.45 ± 0.05	1.65 ± 0.28 **	1.81 ± 0.30 **^,#^
LVFS (%)	52.8 ± 2.1	53.0 ± 1.6	43.4 ± 5.1 **	37.3 ± 4.3 **^,#^
HR (bpm)	499 ± 37	520 ± 44	478 ± 46	509 ± 25

IVSd, interventricular septum diameter; LVPWd, left ventricular posterior wall diameter; LVEDd, left ventricular end-diastolic dimension; LVESd, left ventricular end-systolic dimension; LVFS, left ventricular fractional shortening; HR, heart rate. Data are presented as mean ± SD from 10 to 15 mice in each group. ** *p* < 0.01 *vs.* sham-operated mice, and ^#^ *p* < 0.05 *vs.* WT I/R mice.
